# Advancements and Challenges in Preimplantation Genetic Testing for Aneuploidies: In the Pathway to Non-Invasive Techniques

**DOI:** 10.3390/genes15121613

**Published:** 2024-12-17

**Authors:** Ana del Arco de la Paz, Carla Giménez-Rodríguez, Aikaterini Selntigia, Marcos Meseguer, Daniela Galliano

**Affiliations:** 1 IVIRMA Global Research Alliance, IVI Foundation, Instituto de Investigación Sanitaria La Fe (IIS La Fe), 46026 Valencia, Spain; 2 IVIRMA Global Research Alliance, IVIRMA Valencia, 46015 Valencia, Spain; 3 IVIRMA Global Research Alliance, IVIRMA Rome, 00169 Rome, Italy

**Keywords:** preimplantation genetic testing for aneuploidy, embryo biopsy, non-invasive preimplantation genetic testing, artificial intelligence, time-lapse

## Abstract

The evolution of preimplantation genetic testing for aneuploidy (PGT-A) techniques has been crucial in assisted reproductive technologies (ARTs), improving embryo selection and increasing success rates in in vitro fertilization (IVF) treatments. Techniques ranging from fluorescence in situ hybridization (FISH) to next-generation sequencing (NGS) have relied on cellular material extraction through biopsies of blastomeres at the cleavage stage on day three or from trophectoderm (TE) cells of the blastocyst. However, this has raised concerns about its potential impact on embryo development. As a result, there has been growing interest in developing non-invasive techniques for detecting aneuploidies, such as the analysis of blastocoel fluid (BF), spent culture medium (SCM), and artificial intelligence (AI) models. Non-invasive methods represent a promising advancement in PGT-A, offering the ability to detect aneuploidies without compromising embryo viability. This article reviews the evolution and principles of PGT-A, analyzing both traditional techniques and emerging non-invasive approaches, while highlighting the advantages and challenges associated with these methodologies. Furthermore, it explores the transformative potential of these innovations, which could optimize genetic screening and significantly improve clinical outcomes in the field of assisted reproduction.

## 1. Introduction

Chromosomal aneuploidy is the most frequent genetic abnormality and a main factor contributing to congenital birth defects, implantation failure, and spontaneous miscarriages [1,2]. Under normal conditions, diploid cells contain 23 pairs of chromosomes, a state known as euploidy. However, in cases of aneuploidy, this number is altered, as in monosomy, where cells contain 45 chromosomes, or in trisomy, where they contain 47. These aneuploidies are usually the result of errors during meiosis in the process of gamete formation, whether female or male, where chromosomes or chromatids do not segregate properly. Most of these errors occur during maternal meiosis (~90%), especially during meiosis I, while paternal errors are less common [3]. Factors such as the greater fragility of oocytes during chromosomal recombination and reduced cell cycle control in women contribute to a higher incidence of aneuploidies in embryos [1].

An important factor in the rate of aneuploidies is maternal age. The prevalence of aneuploidies shows a progressive increase with advancing maternal age. In women under 35, the prevalence is relatively low, reaching its lowest point between the ages of 26 and 30, with rates of 20–27%. In contrast, the highest prevalence is observed in women over 42, where aneuploidy rates exceed 80% [4,5].

The implementation of preimplantation genetic testing for aneuploidy (PGT-A) has enabled a more precise selection of euploid embryos, thereby improving the success rates in assisted reproductive technology (ART) [6]. This technique allows the selection of the embryo with the highest implantation potential by evaluating chromosomal anomalies, as well as hereditary conditions, in women of advanced age, with recurrent miscarriage, or recurrent implantation failure [7].

**Figure 1:**
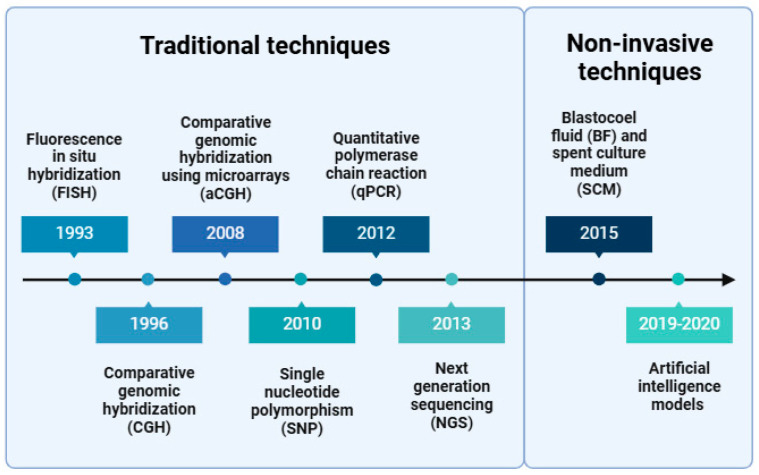
Evolution of PGT-A techniques.

The development of PGT-A techniques has evolved significantly since their inception, allowing for a notable improvement in the precision and scope of genetic testing performed on human embryos prior to uterine transfer (Figure 1).

To perform these techniques, an embryo sample must be obtained through biopsy, a technique that has evolved from its initial applications to enhance precision and minimize impact on embryonic development. These biopsies can be conducted at different embryo stages, such as in polar bodies, blastomeres, and trophectoderm (TE) [8].

### 1.1. Traditional Methods in PGT

#### 1.1.1. FISH

**Figure 2:**
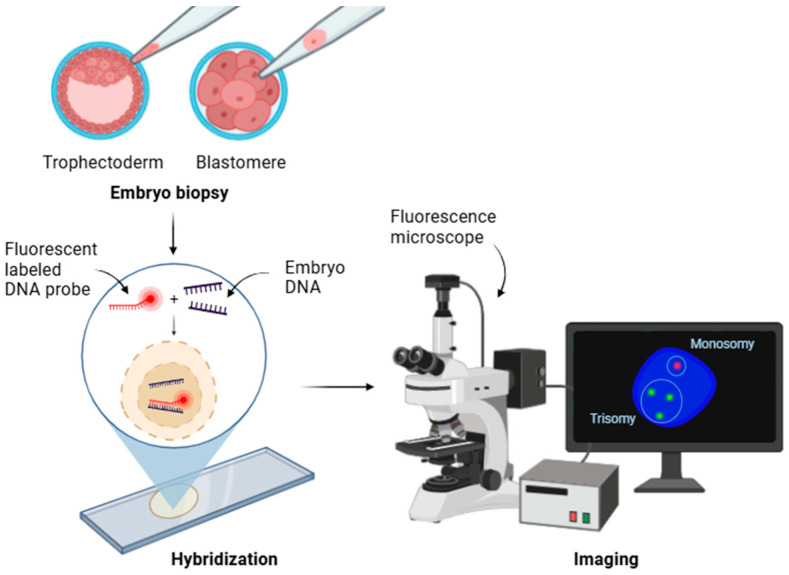
Overview of FISH methodology for chromosomal analysis.

The first generation of PGT was based on fluorescence in situ hybridization (FISH), which was introduced in 1993 using blastomere biopsy [9]. This method uses specific DNA probes labeled with different fluorochromes, which, once hybridized with the sample, enable the observation of results through fluorescence microscopy [10] (Figure 2). In a FISH protocol, the analysis is limited to a range of 5 to 12 probes during 2–3 cycles of hybridization, which primarily focuses on identifying the most common aneuploidies, such as those involving chromosomes 13, 15, 16, 18, 21, 22, and the sex chromosomes [11], which are frequently associated with spontaneous miscarriage, leaving a significant number of anomalies in other parts of the genome undetected. The accuracy of this technique is affected by the overlap, splitting, and diffusion of signals, as well as by probe inefficiency [12,13,14].

Due to the lack of clinical benefits and evidence of disappointing clinical experiences, its use was discouraged [15]. Nonetheless, FISH remains a relevant technique in specific clinical contexts, particularly for diagnosing unbalanced chromosomal abnormalities, such as Robertsonian and reciprocal translocations. This cost-effective method can be applied to a polar body, blastomere, and trophectoderm (TE) biopsies, offering versatility in its use across various stages of embryonic development [16].

**Figure 3:**
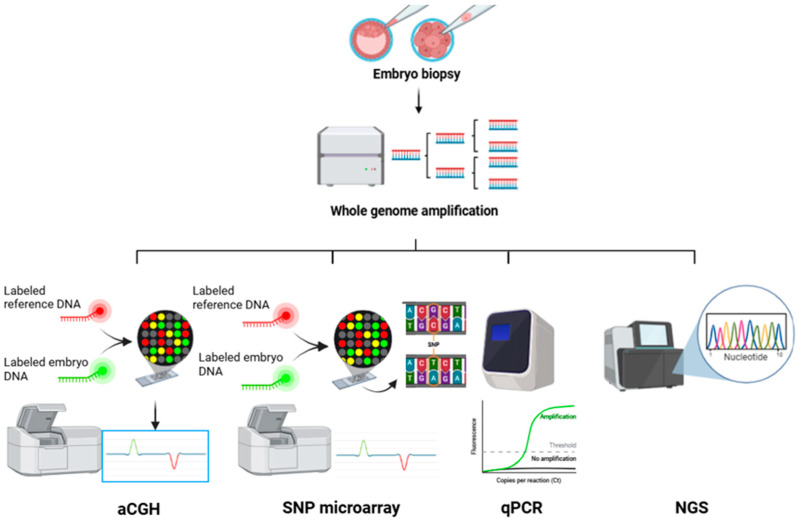
Overview of traditional PGT-A techniques following the introduction of WGA.

Since the amount of DNA in biopsied cells is limited, a key development was whole genome amplification (WGA) [17]. WGA is an established technique to amplify the entire genome from a single cell, generating sufficient DNA for detailed analysis of the 24 chromosomes [18]. Once DNA is amplified, it can be analyzed using various techniques such as comparative genomic hybridization arrays (aCGH), single nucleotide polymorphism (SNP) arrays, or next-generation sequencing (NGS) [12] (Figure 3).

#### 1.1.2. CGH and aCGH

The FISH technique was initially replaced by comparative genomic hybridization (CGH), a molecular cytogenetic technique developed in 1992 [19] to detect chromosomal anomalies in tumors and first applied to blastomeres in 1996 [20]. This methodology identifies losses or gains of DNA fragments that have been previously amplified by WGA. It compares the fluorescence emitted by normal DNA labeled with a fluorochrome to that of blastomere DNA, also labeled with fluorochromes, both hybridized onto a metaphase spread [21]. A key limitation of CGH is its speed, as obtaining results requires several days. This delay complicates its use in clinical settings where rapid diagnosis is essential for timely embryo transfer [22]. Furthermore, it has a low resolution, which led to its replacement by aCGH, known for its accuracy and high specificity [12].

The CGH technique using microarrays (aCGH) emerged in 1997 as an evolution of the original technique, where instead of using metaphase chromosomes, specific probes for chromosomal regions are employed on a glass surface [23], automating the fluorescence evaluation process, and reducing the hybridization time [22].

A validation study showed that using aCGH for single embryo selection in PGT-A significantly improved implantation and ongoing pregnancy rates, reaching 69% compared to 42% in the control group. This highlights its effectiveness in embryo selection during in vitro fertilization (IVF) [24].

Typically, blastocyst vitrification and frozen embryo transfer are required when applying CGH. However, it has been demonstrated that the time needed for the procedure can be reduced to 9 h, thus allowing for fresh transfers. Furthermore, the reproductive outcomes obtained are comparable to those of conventional aCGH and NGS [25].

This PGT-A technique, while allowing for rapid and accurate detection of aneuploidies, presents several limitations. It cannot identify haploid or polyploid embryos, balanced chromosomal rearrangements, or individual mutations. Additionally, it requires advanced technology, which increases both the complexity and cost of the methodology. aCGH is limited to identifying whole chromosome aneuploidies or unbalanced translocations, such as those associated with parental Robertsonian and reciprocal translocations, and limited mosaicism, which is detected with lower sensitivity when compared to more advanced techniques like NGS [12,26,27,28].

#### 1.1.3. SNP

One PGT-A technique based on microarrays is the SNP microarray, which is based on the identification of small variations in DNA called SNPs, representing a variation in a single base pair in an organism’s DNA sequence, distributed throughout the genome. SNP microarray compares these variations with a reference genome, typically derived from both maternal and paternal sources [12]. These microarrays typically evaluate approximately 300,000 SNPs in a single genetic analysis [29]. Unlike other methods such as CGH, where the embryo DNA and reference DNA are hybridized simultaneously, in the SNP microarray, both samples are hybridized separately and then compared. This not only allows for the detection of aneuploidies but also facilitates the identification of the parental origin of any chromosomal anomaly. The technique assesses two key aspects: first, the alleles detected in the embryo are compared with those of the parents to determine which chromosomes were inherited. Second, the intensity of the fluorescence signals is measured; a higher intensity indicates an excess of genetic material (trisomy), while a lower intensity suggests a loss of chromosomes (monosomy) [22].

The use of this methodology, combined with embryo vitrification and frozen embryo transfer, has demonstrated promising reproductive outcomes, with implantation rates of approximately 65% per blastocyst transferred and live birth rates of 73% per embryo transferred, highlighting its strong reliability and suitability for implementation in clinical practice [30].

SNP array can detect whole chromosome aneuploidies, and unbalanced translocations, determine the parental origin of monosomies and trisomies, uniparental disomy, and polyploidy, as well as duplications and deletions with high resolution [21,31].

However, this technique also has limitations. The analysis time is lengthy, potentially extending up to 72 h. In addition, it has a limited ability to detect small structural aberrations, those smaller than 5 Mb, and it is a complex and costly process [21,32].

#### 1.1.4. qPCR

Quantitative polymerase chain reaction (qPCR) is a nucleic acid amplification methodology that allows for the identification of abnormalities in complete chromosomes through the quantification of the present copies of each analyzed chromosome. This procedure is based on the comparison of three to four locus-specific amplicons along each chromosome, enabling an efficient analysis of the 23 pairs of chromosomes [29].

By employing multiplex PCR at 96 loci (96-plex), followed by high-throughput qPCR, this approach streamlines the process, reducing the number of loci required to assign chromosome copy numbers accurately [33,34].

This technique presents various advantages, such as low cost, high efficiency, and short turnaround time, making it suitable for fresh embryo transfers within the same cycle.

However, the use of only 96 probes imposes limitations on its ability to detect complex genomic alterations such as segmental aneuploidies, mosaicism, and unbalanced translocations, especially when these variations occur outside the targeted regions [30,35].

Despite these limitations, the use of qPCR as a PGT-A technique, which analyzes the entire genome, has been shown to improve implantation and delivery rates, as observed by Werner et al. [34], highlighting its importance and practical applicability.

#### 1.1.5. NGS

Currently, the most widely used technique is NGS [36]. To perform this technique, a biopsy is required, which can be optimized through assisted hatching or laser manipulation. Between five and ten cells from the blastocyst’s TE are collected on days five and seven post-fertilization; these cells are destined to form the placenta. This approach offers advantages over extracting one or two blastomeres at the cleavage stage, as it reduces the number of cells taken from the embryo, increases the amount of DNA available for analysis, decreases the risk of technical errors, and allows for the detection of mosaicism in the biopsy [17,37].

The NGS technique provides higher resolution compared to previously described techniques, resulting in improved clinical outcomes. NGS uses low-coverage sequencing; that is, the DNA of interest is fragmented into small segments labeled with a specific sequence. These fragments are read and compared with a reference genome, grouped into “bins”, and generate a karyotype profile encompassing all 24 chromosomes of the embryo [12,17]

Although this method analyzes small fragments, it is precise in quantifying the chromosomal copy number present in the sample of interest, allowing for the detection of monosomies and trisomies. Data obtained on nullisomies, monosomies, disomies, trisomies, and tetrasomies indicate the chromosomal copy number: 0, 1, 2, 3, and 4, respectively. Intermediate values, such as 2.5, suggest mosaicism, meaning a mixture of cells with different chromosomal copy numbers within the embryo. Most laboratories identify mosaicism in the range of 20% to 80% in karyotype profiles. Values below 20% are considered normal noise and do not indicate mosaicism [17].

NGS, although considered a more effective alternative compared to other techniques due to its higher resolution and ability to detect mosaics, also presents certain limitations. This PGT-A technique cannot detect balanced chromosome translocations, as there is no imbalance in the total amount of DNA. [38]. In addition, the use of NGS can be costly if it is not optimized by having enough samples in each sequencing run [39].

### 1.2. Traditional PGT-A Techniques Challenges

The primary limitation of PGT-A techniques is the need to perform an embryo biopsy to carry out chromosomal analysis. The removal of embryo cells could compromise its viability [40].

Another significant limitation of PGT-A is embryonic mosaicism, which refers to the presence of different cells with distinct chromosomal compositions within the same embryo [41]. Since trophectoderm (TE) biopsy followed by chromosomal analysis using NGS is the standard PGT-A technique, this phenomenon presents a major challenge, as it may not accurately reflect the global chromosomal status of the embryo, leading to false positives (discarding viable embryos) or false negatives (transferring non-euploid embryos). This technical aspect directly compromises the reliability of the technique and may impact implantation rates and live birth rates (LBR) [42,43].

Another relevant challenge is the lack of conclusive evidence regarding the clinical benefits of PGT-A. This technique was not clinically validated or certified by any professional organization before its routine implementation in assisted reproduction clinics. Although the use of PGT-A increased between 2014 and 2017, LBR did not show significant improvement [44]. This technique has only demonstrated an increase in pregnancy rates in women over 35 years old or those with repeated IVF failures, but not in the general population [45]. In young women, no significant increase in LBR has been demonstrated in PGT-A cycles compared to cycles without PGT-A, so it should not be recommended routinely but rather restricted to specific cases where a clear benefit has been evidenced [46].

Furthermore, the high cost associated with PGT-A limits its accessibility, benefiting mainly those with greater financial resources. In healthcare systems where PGT-A is not covered by insurance, the cost can be excessive, as seen in the United States, where it can reach up to USD 12,000, thereby excluding demographic groups with fewer resources [46].

Addressing these limitations is crucial to optimizing the use of PGT-A and ensuring that its benefits translate into tangible improvements for a broader range of patients.

### 1.3. Emerging Non-Invasive Techniques in PGT

Historically, PGT-A has involved the invasive removal of one or more cells from the embryo, which has raised concerns about potential negative effects on embryonic development, potentially compromising its quality and viability [47]. Additionally, PGT-A is a costly and labor-intensive procedure that requires a specialized laboratory and trained embryologists to minimize the number of embryo biopsies and reduce the risk of damaging or compromising the embryo’s viability and cryopreservation [48].

**Figure 4:**
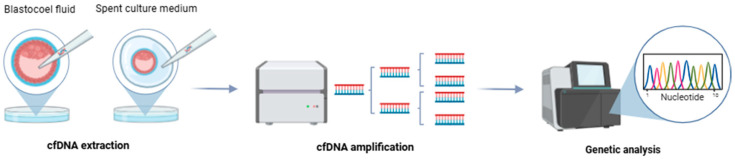
Overview of non-invasive PGT-A methods.

These limitations, along with the discovery of cell-free DNA (cfDNA) in blastocoel fluid (BF) and spent culture medium (SCM), have generated interest in non-invasive alternative techniques [49] (Figure 4).

There are three non-invasive PGT for euploidy assessment: analysis of BF, SCM, and the use of artificial intelligence (AI) algorithms associated with embryo imaging and clinical parameters.

Regarding cfDNA detected in the BF and in the SCM, it may have various origins, with apoptosis being the most studied. Through this process, defective or excess cells are eliminated, which can release fragmented DNA into the perivitelline space or the BF.

It has been observed in mosaic chimeric mice that aneuploid cells from the TE persist, while those from the inner cell mass (ICM) are expelled into the BF. This results in a higher proportion of aneuploid cells in the fluid, which may lead to false positives for aneuploidy [49].

The identification of DNA fragments ranging from 160 bp to 220 bp in length and mitochondrial DNA in the BF supports this hypothesis [50]. Additionally, the presence of mRNA encoding pro-apoptotic genes has also been detected, which may be involved in communication between embryo cells, reinforcing the hypothesis of induced cell death. These RNA molecules act freely or within extracellular vesicles, the presence of which has been recently confirmed [36,50].

Variations associated with the ploidy status of the embryo have recently been identified. In euploid embryos, genes such as CASP7 and MCL1 have been associated with successful implantation, while genes such as TNFRSF25 and BCL2L11, among others, are found in embryos that do not implant. Lal et al. (2022) observed a significantly higher expression of apoptotic genes in implanted embryos compared to those that did not implant [51].

#### 1.3.1. cfDNA in BF

Palini et al. (2013) was the first to identify DNA in BF [52]. Blastocentesis involves aspirating the BF, which is the fluid-filled cavity of the embryo at the blastocyst stage. Using an ICSI pipette, the trophoblast is penetrated on the side opposite to the location of the ICM, thereby causing the embryo to collapse [49]; thus, this method is not completely non-invasive [12].

The collapse and re-expansion of the embryo are routine processes in in vitro fertilization (IVF) laboratories, so aspirating this fluid should not be harmful to the embryo [53].

This BF contains fragments of the embryo’s DNA, which can be analyzed for the detection of aneuploidies or other chromosomal abnormalities, providing valuable information for embryo selection without damaging the embryo’s cells as a biopsy would.

Although this technique requires skill, it is less complex and invasive than a biopsy of embryonic cells. However, it presents several limitations.

The main difficulty lies in the inconsistent amplification of DNA, as the amount available is extremely low, ranging from 0.3 nL to 1 µL. The variability of this amplification ranges from 27% to 87% [53], reflecting the inefficiency of current amplification protocols for blastocoelic DNA. This inconsistency could be due to several reasons, such as the absence of DNA in the BF, the small quantity and quality of DNA present, as well as technical difficulties in handling such small volumes. Additionally, the degraded nature of the DNA can complicate amplification [49].

The amount of DNA in the blastocoel largely depends on the state of the embryo and the time elapsed since fertilization. Evidently, expanded blastocysts are more likely to have greater availability of DNA [54]

Several studies have been conducted to evaluate the effectiveness of this technique as a PGT method, yielding variable results. Gianaroli et al. (2014) [55] and Magli et al. (2016) [54] reported favorable outcomes, with amplification rates of 76.5% and 82% and concordance rates with PGT-A through TE biopsies of 82% and 81%, respectively.

However, other studies did not achieve such encouraging results. Perloe et al. (2013) [56], Tobler et al. (2015) [57], Tšuiko et al. (2018) [58], and Capalbo et al. (2018) did not observe favorable outcomes. Perloe et al. (2013) reported an amplification rate of only 28.1% and a concordance rate with TE biopsy of 33.3%. Tobler et al. (2015) found a 52% discordance between BF samples and biopsies of both the ICM and TE. Tšuiko et al. (2018) [58] observed a 40% concordance between ICM and TE samples, while Capalbo et al. (2018) [59] reported a concordance rate of 37.5% along with an amplification rate of 34.8%. Finally, Magli et al. (2018) [60] observed a concordance of 93.6% between BF samples and TE samples.

Due to the variability observed across different studies regarding amplification and concordance between BF samples and TE biopsies, blastocentesis does not yet appear to be a reliable technique for PGT-A.

#### 1.3.2. cfDNA in SCM

An alternative for minimally invasive analysis is the detection of cell-free DNA in SCM. The first identification of DNA in this medium was reported in 2014 [61]. Subsequently, it has been demonstrated that culture medium samples from embryos contain a greater amount of DNA compared to BF samples, suggesting it may serve as a better source of genetic material and as a truly non-invasive technique [62].

This method is based on the release of DNA fragments into the culture medium due to cellular processes such as apoptosis, allowing DNA collection without harming the embryo. A culture period of 24 to 48 h is sufficient to obtain useful samples [63].

This technique has generated significant interest because it enables broader genetic analysis; cfDNA could originate from both the TE and the ICM, including fragments from both viable embryo cells and discarded aneuploid cells [64].

In 2020, a systematic review was conducted to evaluate the efficacy of this technique [65]. The results showed that DNA detection in SCM ranged from 6.7% [62] to 100% [61,66,67,68,69]. DNA amplification rates also varied considerably between studies, with a significantly higher detection-amplification rate in SCM of blastocysts compared to cleaved embryos [61,62,67,70,71].

The results of genetic analysis using SCM were comparable to those obtained with invasive techniques, including pregnancy and live birth rates. Regarding ploidy, a wide variability has been reported in the concordance between SCM and TE biopsies, ranging from 15.4% [68] to 100% [62,66,69]. These differences could be attributed to factors such as mosaicism.

Notably, the studies showing higher overall concordance rates, between 85.7% and 100%, were those where the SCM was collected from blastocysts developing over 24–48 h [69,72].

Several studies have identified exogenous and maternal contamination in SCM samples [71,73,74]. The presence of extra-embryonic DNA complicates the interpretation of cfDNA results, representing one of the main challenges in the accuracy of this technique. Maternal DNA could come from components of the oocyte, such as cumulus cells and the corona radiata, even after being denuded in IVF techniques. Additionally, extra-embryonic DNA may originate from the media used in ART, additives, or as a result of manipulation by the embryologist [71].

Due to the high heterogeneity in study designs evaluating this technique, determining its efficacy is challenging. Therefore, before considering its implementation in assisted reproduction clinics, new multicenter studies with optimized procedures and larger sample sizes should be designed [65].

#### 1.3.3. AI Algorithms for Testing Ploidy

Additionally, another non-invasive alternative is based on the analysis of embryo images using AI algorithms. AI has emerged as a revolutionary tool in embryo selection. This branch of computer science aims to emulate intelligent behaviors associated with human beings, such as thinking and problem-solving [63].

In the field of assisted reproduction, AI has begun to be applied to enhance the classification and non-invasive selection of embryos. It has been used in various areas, from annotating embryonic development to predicting ploidy and the outcomes of ART [75].

Traditionally, the evaluation of embryo quality has relied on morphological criteria observed at specific stages of development. However, there is considerable interobserver and intraobserver variability, as this is a subjective method dependent on the skill of the embryologist and the developmental stage being evaluated [76].

AI offers a more efficient and precise alternative by automating embryo selection through advanced machine learning algorithms and image analysis. It relies on machine learning algorithms and convolutional neural networks (CNNs) that can analyze large volumes of data and detect complex patterns that may be overlooked by embryologists [77]. These algorithms process images of the embryo captured using time-lapse technology, used for the first time in 1997 [78], which allows for continuous observation of embryos from the earliest stages to the blastocyst stage without needing to remove them from the incubator, thereby avoiding changes in culture conditions [79].

This approach captures the complete development of the embryo in real time, providing information about cell division timings, blastocyst expansion, and other key morphokinetic parameters [80] that algorithms can correlate with implantation probability and the development of a viable pregnancy [81], improving IVF outcomes [82,83,84]

This tool has evolved from being a simple observation tool to one of selection and prediction. Time-lapse imaging systems allow the acquisition of morphokinetic parameters in addition to a morphological evaluation of the embryos [85]. Embryo selection algorithms use these morphokinetic data, with their nomenclature standardized according to guidelines such as those proposed by Ciray et al. [86].

Some time-lapse systems include decision-support tools for selecting the optimal embryo. In the case of Vitrolife, KIDScore D5™ version 1 and 2 (EmbryoViewer software; Vitrolife) is used, an algorithm that classifies embryos based on their implantation potential through automatic annotations [87]. The latest version of this algorithm (version 3) evaluates the number of pronuclei, division times (t2 to t5), and the quality of the ICM and TE, assigning a score to the embryo on a scale from 1 to 9.9, representing implantation probability from low (1) to high (9.9). Additionally, it has been recently observed that this latest version of the algorithm tends to assign a higher score to euploid embryos compared to aneuploid ones [88].

There are also instant, objective, and automated web applications aimed at supporting the decision-making process when selecting an embryo within a cohort. An example is Life Whisperer Genetics (LWG) [89], which evaluates morphological characteristics associated with ploidy status. LWG identifies the highest-quality embryo as euploid in 82% of cohorts [90].

Another well-known AI model is iDAScore, developed by Vitrolife, which evaluates blastocyst quality automatically using deep learning. This model classifies blastocysts by assigning them a score, where a low score is associated with delayed development, compaction, and expansion of the blastocyst [91]. In addition to a significant correlation between iDAScore and implantation, ongoing pregnancy, miscarriage, and live birth, a similar relationship has been observed with euploidy, with higher scores assigned to euploid blastocyst [92,93,94,95].

##### AI Models for Ploidy Prediction Studies

Several researchers have developed specific models to address ploidy prediction using AI. The Embryo Ranking Intelligent Classification Algorithm (ERICA), developed by Chávez et al. [96], is one of the pioneering approaches in AI applied to high-resolution static embryo image analysis for ploidy assessment through CNNs. Its development involved training the model with a large dataset composed of blastocyst images with known prognoses, along with clinical data, allowing it to learn to identify patterns associated with ploidy status and predict implantation potential.

Embryos were classified as “good prognosis” if they were euploid and implanted, or “poor prognosis” if they were aneuploid and did not implant. ERICA’s predictive capability was compared to both random assignment of prognosis labels and classification performed by experienced embryologists, with the model demonstrating statistically significant superiority over both [96].

Overall, ERICA achieved an accuracy of 0.70 in ploidy prediction, with a positive predictive value of 0.79. Additionally, in 78.9% of cases, it ranked a euploid embryo in the top position, and in 94.7% of instances, it included at least one euploid embryo within the top two rankings, with an average ranking time of under 25 s [96].

In 2022, Diakiw et al. [90] developed a new model to predict the ploidy status of day five blastocysts from static images captured via optical microscopy. Over 15,000 images with associated clinical outcomes were used, of which 5050 were selected for model training. These images were linked to genetic data obtained through PGT-A, indicating whether the embryo was euploid or aneuploid.

The model achieved an accuracy of 65.3% in predicting euploidy in a test image set, with a sensitivity of 74.6%, which increased to 77.4% after filtering out low-quality images. Additionally, the model generated an AI score for each embryo, revealing that embryos with a high score (9.0–10.0) were twice as likely to be euploid compared to those with a low score [90].

Within the same embryo cohort, the probability of the top-ranked embryo being euploid was 82.4%, and 97% when considering at least one of the two top-ranked embryos. This model proved to be 26.4% more effective than random ranking and 13–19% more effective than the Gardner grading system [90].

In 2023, Barnes et al. designed STORK-A [48], a model developed to predict ploidy non-invasively. It utilized a retrospective dataset consisting of 10,378 static images of blastocysts taken 110 h post-fertilization, along with morphokinetic parameters, morphological assessments, maternal age, and ploidy status as determined by PGT-A. This model demonstrated an accuracy of 69.3% in predicting euploid versus aneuploid embryos, with a positive predictive value of 76.1% and a negative predictive value of 62.1%.

STORK-A was also the first model to analyze complex aneuploidies, successfully classifying complex aneuploid embryos versus euploid and simple aneuploid embryos with an accuracy of 74% and distinguishing complex aneuploidy from euploidy with 77.6% accuracy [48].

When evaluated with two external datasets (Weill Cornell Medicine and IVI Valencia), it achieved similar accuracy to the training dataset, with AUC values of 0.702 and 0.715, respectively [48].

In 2021, Lee et al. [97] used a retrospective dataset composed of 690 embryo videos in developmental stages up to the blastocyst stage associated with PGT-A results. For the training phase, the Two-Stream Inflated 3D ConvNet (I3D) model was used with 80% of the videos. Both RGB (red, green, and blue) and optical flow images were used and the combined model of both reached an area under the curve (AUC) of 0.74 for ploidy prediction. Although this model outperforms other classification systems such as KIDScore, it does not include maternal or clinical factors, which could improve accuracy.

Yuan et al. [98] developed a model based on time-lapse imaging to predict ploidy and live birth rate. For this purpose, a dataset of 1396 blastocysts analyzed via PGT-A was used, incorporating morphokinetic, morphological, and clinical parameters from 403 patients. This model, employing morphokinetic characteristics such as cell division timings along with the KIDScore and Gardner grading, achieved an AUC of 0.879 in predicting ploidy. Furthermore, the results indicated that euploidy was associated with morphokinetic and morphological parameters, though no significant correlation was found for the live birth rate.

The same year, Huang et al. [99] created the EPA (Euploid Prediction Algorithm). To do so, they used a retrospective dataset consisting of 469 PGT-A cycles and 1803 blastocysts, with images taken on days five to six post-fertilization using the time-lapse system of the Embryoscope Plus. This model utilizes the 3D-ResNet50 convolutional neural network to extract features from image sequences and predict ploidy. The data included morphokinetic parameters and morphological characteristics of the blastocysts, as well as clinical information from the patients. EPA achieved an AUC of 0.80 in the test set. Furthermore, in the external validation performed with an additional 523 blastocysts from 155 PGT cycles, the model maintained promising results in its ability to discriminate ploidy, suggesting its potential clinical applicability as a non-invasive tool for selecting euploid embryos.

## 2. Discussion

Invasive traditional techniques have been fundamental for PGT and aneuploidy detection, significantly contributing to the success rates of ART. Although effective [100] these methods require the biopsy of cells from blastomeres on day three or from TE cells at the blastocyst stage [101], which may potentially impact embryo development, viability, and implantation success [102,103,104]. This has led to a focus on developing non-invasive techniques that can provide genetic information without compromising embryo integrity.

Among non-invasive techniques, cfDNA analysis from the BF and SCM has shown promising results, allowing for genetic analysis without direct intervention on the embryo. However, these methods face limitations, such as potential DNA contamination with non-embryonic extracellular material [54,70,105], which may affect the reliability of these results.

AI has emerged as an innovative tool to address these challenges. AI-based models allow the analysis of morphokinetic and morphological data from embryos obtained through time-lapse imaging systems [106]. These models employ deep learning algorithms to examine development patterns and cell division dynamics, identifying those associated with euploid embryos.

The use of AI also presents significant challenges. One is the need for large volumes of high-quality data, ideally from multicenter sources, to train and validate models robustly [77]. The lack of standardization in imaging capture systems and incubation protocols across laboratories can introduce variability in the data, affecting the precision and generalizability of these models [107]. Furthermore, many current models rely solely on morphokinetic and morphological parameters, excluding clinical and maternal factors that may influence the chromosomal status and embryo viability [97]; including these additional data could significantly improve model performance [108].

Another limitation is the limited capacity to predict complex chromosomal statuses, such as mosaicism, where the embryo exhibits cells with different chromosomal compositions [77].

Given these unresolved limitations, using AI-based models for aneuploidy prediction is primarily recommended when traditional PGT-A techniques are inaccessible, there are additional embryos available in the cohort, or when used alongside PGT-A to prioritize embryos for biopsy [85]

In the future, enhancing the precision and applicability of these models in laboratories will be essential. Algorithm optimization integrating clinical and patient-specific parameters, alongside imaging data, is required. Additionally, conducting multicenter studies with larger sample sizes to validate these models across diverse populations and contexts is crucial, enabling the development of more robust and generalizable algorithms.

## 3. Conclusions

In conclusion, traditional PGT-A techniques require embryo biopsy, raising concerns about their potential impact on development. Emerging non-invasive techniques that preserve embryo integrity offer a promising approach to improving embryo selection. Methods such as the genetic analysis of cell-free DNA in blastocoel fluid (BF) and spent culture medium (SCM), as well as artificial intelligence (AI) models applied to ploidy, have shown encouraging results. However, these techniques face several challenges. For cell-free DNA, it is crucial to optimize detection sensitivity and minimize the risk of external contamination, while for AI models, large-scale clinical implementation still encounters limitations that need to be addressed. With continuous advancements in detection precision and clinical validation of these techniques, they could become a reliable alternative for embryo selection, significantly enhancing ART outcomes.
